# Intrauterine Upper Limb Ischemia: An Unusual Presentation of Fetal Thrombophilia—A Case Report and Review of the Literature

**DOI:** 10.1155/2013/670258

**Published:** 2013-10-08

**Authors:** Samer Abdelrazeq, Abdullatif Alkhateeb, Hani Saleh, Haitham Alhasan, Hatem Khammash

**Affiliations:** ^1^Department of Pediatrics, Al-Quds University, Makassed Hospital, Jerusalem, Palestine; ^2^Department of Pediatrics and Neonatology, Al-Quds University, Makassed Hospital, Jerusalem, Palestine; ^3^Department of Pediatrics, Hemato-Oncology Unit, Augusta Victoria Hospital, Jerusalem, Palestine; ^4^Department of Vascular Surgery, Makassed Hospital, Jerusalem, Palestine; ^5^Neonatology Department, Makassed Hospital, P.O. Box 22110, Mount of Olives, Al-Tour, Jerusalem, Palestine; ^6^Pediatric Department, Al-Quds University, P.O. Box 22110, Mount of Olives, Al-Tour, Jerusalem, Palestine

## Abstract

Upper limb ischemia presenting in neonatal period is extremely rare. Moreover, presenting newborn with evidence of intrauterine upper limb vascular occlusion is even rarer. It needs prompt intervention to restore perfusion and avoid morbidity. We present a newborn with right upper limb brachial artery thrombosis causing ischemia that was noted at birth and appeared later to be homozygous for factor V Leiden and glycoprotein IIIa with no other identifiable risk factors. In this report, we present the case, its successful medical management, proper counseling, and review of the literature. We recommend investigating the neonates and their parents for thrombophilia mutations when they present with unusual vascular occlusion site as newborns.

## 1. Case Presentation

Our case is a full term infant product of nonconsanguinity marriage, uneventful pregnancy born by urgent Cesarean section due to failure to progress, cephalic presentation. Birth weight was 3700 grams (appropriate for gestational age) and Apgar Score was 9 and 10 in first one and five minutes, respectively. The mother is 25 years old, gravida 1, para 0, and abortion 0. He has an uneventful prenatal history and a normal antenatal ultrasound with no history of maternal diabetes mellitus or preeclampsia. Family history is unremarkable neither for early nor for late thromboembolic phenomena nor for any recurrent abortions. Placenta gross examination was normal.

**Figure 1:**
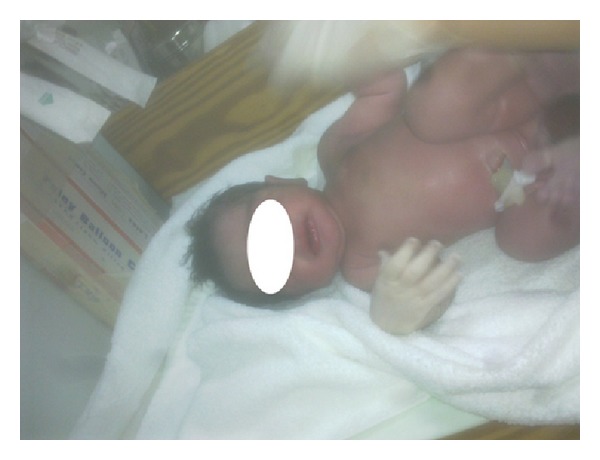
Our patient at age of 6 hours showing pallor and cyanosis from proximal one third of arm to all hands and fingers.

The patient was delivered at a maternity unit and noticed at delivery to have a pale right upper limb so he was admitted to the Neonatal Intensive Care Unit (NICU) at our hospital. On examination it was found that he had asymmetrical moro reflex, right upper limb coldness, pallor, and cyanosis from proximal one-third of arm to all hands and fingers (Figure 1). The patient could move his arm spontaneously with intact grasp reflex and pain sensation. Radial, ulnar, and brachial pulses could not be felt. Capillary refill time was prolonged (5 seconds). Pulse oximeter did not read Oxygen saturation on the affected limb, but was normal on the other limbs. The rest of the examinations were normal including growth parameters.

Investigations done for our patient showed normal complete blood count (CBC) values including platelet and hemoglobin. C-Reactive Protein, ESR, and Coombs' test were negative and so was the blood culture. Coagulation profile and homocysteine level were normal [1–5]. 

**Figure 2:**
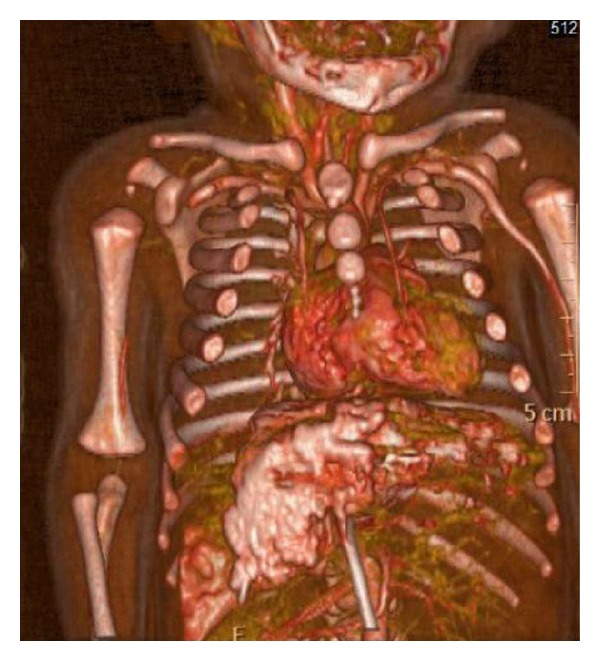
Three-dimensional view on computed tomography angiography showed right axillary artery occlusion from its first part till brachial artery in mid arm with no evidence of collaterals, consistent with a diagnosis of right axillary artery thrombosis.

X-ray of chest and upper limbs showed no cervical rib or fractures. Echocardiography showed no evidence of congenital heart disease with normal anatomical branches of the aorta. Doppler ultrasound of the right upper limb showed evidence of no flow in the brachial artery, while venous flow was normal. Computed tomography angiography (CTA) for aorta and its branches including right upper limb vasculature was done at age of 18 hours and revealed that the right axillary artery was occluded from its first part till brachial artery in mid arm with no collaterals (Figure 2: three-dimensional view on computed tomography angiography) consistent with a diagnosis of right axillary artery thrombosis that is causing upper limb ischemia. Otherwise, aorta, inferior vena cava, umbilical, and renal vessels were normal. Transfontanel ultrasound was negative for any hemorrhage. Blood fibrinogen level was low, 152 mg/dL (range 200–400 mg/dL), and D-dimers level was 890 ng/mL (normal < 200 ng/mL) supporting a recent forming thrombus.

Blood levels for protein C, protein S, antithrombin activity, antiphospholipid antibodies, anticardiolipin, concentrations of clottable fibrinogen, activities of coagulation factors VIIIC and XII, and lipoprotein (a) were not done due to unavailability [5]. Extensive work-up for thrombophilia mutations for our patient and his parents was made [6]. DNA-based assays for ACE I/D, PAI-1 4G/5G, Factor XIII Val34Leu, MTHFR C677T, and prothrombin G20210A were normal [6]. But he was homozygous for factor V Leiden R506Q and homozygous for Glycoprotein IIIa Leu33Pro. This strongly explains the cause of his ischemic occlusion and neonatal presentation especially after a stressful event such as delivery.

Meanwhile, the patient was started on Heparin drip (20 units/kg/hour) for 4 days [6] that adjusted according to target activated Partial Thrombin Time “aPTT” of 2-3x normal with significant improvement and complete recovery. Afterwards, pallor disappeared, capillary refill time normalized, and Oxygen saturation read 98% on both upper limbs. Brachial and radial pulses felt with a good volume. Doppler ultrasound was repeated, and it showed normal arterial flow. Patient then shifted to low-molecular-weight Heparin (Enoxaparin) (2 mg/kg subcutaneously BID [7]) and discharged on that till the age of one month. In addition to that, the patient was put on antibiotics and intravenous fluids initially that were discontinued after ruling out sepsis and achieving full feeding.

Magnetic resonance imaging (MRI) of brain was not done as he had normal neurological exam and no focal neurological evidence. Trans-fontanel ultrasound was satisfactory to rule out hemorrhage before starting anticoagulation.

The patient was readmitted at age of one month and underwent circumcision after stopping Enoxaparin. Then the patient was started on warfarin with target INR 2-3 [8] for one month then shifted again to Enoxaparin with regular followup in a pediatric hematology clinic. Enoxaparin was stopped at age of 16 months. The Patient is now 24-month-old, with normal examinations and no evidence of old or new vascular event, nor evidence of hemorrhage.

Thrombophilia mutation for father revealed that he is heterozygous for Factor V Leiden, Glycoprotein IIIa, PAI-1 4G/5G, and ACE I/D. And for the mother that she is heterozygous for Factor V Leiden, Glycoprotein IIIa, MTHFR, PAI-1 4G/5G, and ACE I/D. The mother did not have a history of hypertension, glucose intolerance, infection, or hypercoagulable status during pregnancy.

Proper counseling for parents was given and they were advised a regular followup with a hematologist and a geneticist. Risk of recurrence and mode of inheritance were clarified. Parents were advised a full detailed followup in next pregnancy to have a prenatal diagnosis for fetal thrombophilia study.

## 2. Discussion

The prevalence of symptomatic neonatal arterial thrombosis is approximately 1 in 20,000–40,000 births and reaches 2.4–6.8 per 1000 neonatal intensive care admissions in some reports [9–12]. Approximately, 90% of cases are iatrogenic and linked to indwelling intraarterial catheters and are rarely described at birth [1–4, 13]. 

Other major risk factors are maternal diabetes or lupus, preeclampsia, sepsis, polycythemia, asphyxia, oligohydramnios, intrauterine growth retardation, significant dehydration, long obstructed labour, and inherited thrombophilia [1, 3–5, 14–18]. Some inherited thrombophilia defects, for instance, Factor V G1691A, prothrombotic polymorphisms, Factor II G2021A, and the homozygous TT genotype of the methylenetetrahydrofolate reductase (MTHFR) C677T polymorphism, are linked to increased risk of neonatal arterial thrombosis [19].

Very few case reports of spontaneous neonatal arterial thrombosis at birth have ever been described in the literature [1, 3, 7, 14, 20–29]. Moreover, presenting newborn with evidence of intrauterine upper limb vascular occlusion is even rarer [14, 20, 23, 24, 27, 28]. The majority of them occur primarily in the large vessels, namely, in aorta mimicking cyanotic heart disease [3, 4, 13, 30, 31] and as renal vein thrombosis [1–3, 27]. Nagai et al. summarized the characteristics and treatments used in previously reported patients with intrauterine extremity thrombosis [20] reporting that only a few case reports achieved a favorable outcome using medical and even surgical intervention.

Moreover, it is obvious that these ischemic insults appear to predominate in the upper limbs, and cases are equally divided between left and right sides and are more predominant in males [20, 21, 32]. Metsvaht et al. stressed on the striking prevalence of male gender among patients with spontaneous neonatal aortic arch thrombosis that might suggest the role of sex-linked inherited factors in the penetrance of disease, as autosomal dominant inheritance has been established for the FVL mutation [33].

Prevalence of thrombophilia markers is increased in children with thrombosis compared with control subjects. Many reports reported the high prevalence (44–81%) of at least one single thrombophilia marker in neonates with vascular thromboembolism [32, 34]. Moreover, having a combination of ≥2 thrombophilia markers makes the risk even more significant [32, 35].

Increased prevalence of Factor V Leiden was observed in some reports on pediatric arterial thromboses and stroke [9, 35–38]. Factor V Leiden [35], which is by far the most frequent inherited prothrombotic condition with a prevalence of about 5–10% in Caucasian population [11, 39–42], is significantly associated with spontaneous and catheter-related thrombosis [15, 32, 34, 43, 44]. It is reported that the risk of spontaneous thrombosis increases 8-fold in the heterozygous carrier and 80-fold in the homozygous patients [29]. Nowak-Göttl et al. [45] reported that 38% of neonates and children with arterial thromboembolism were positive for Factor V Leiden gene mutation, and Hagstrom et al. [46] found that 27% of neonates with arterial stroke had the Factor V Leiden mutation. 

On the other hand, Glycoprotein IIIa homozygosity is a major risk factor for coronary thrombosis and myocardial infarction and an important predictor for sudden cardiac death for middle ages. Moreover, it is an inherited risk that promotes thromboembolic complications of pregnancy and a prognostic factor for early fetal losses. 

Moreover, the combined inheritance of prothrombotic risk factors further increases the risk of early thrombosis [47]. But none of the above-mentioned articles targeted the combined effect of factor V Leiden and Glycoprotein IIIa as risk factors for early thrombosis presenting in neonatal period. 

Clinical features of peripheral arterial occlusion are gathered as the 6Ps: pallor, pulselessness, paralysis, pain, parasthesia, and perishing cold of involved extremity. At least four of the above were elicited in our patient. The clinical presentation varies depending on the site and time of occlusion [14, 20, 21]. In our case, we propose that the time of ischemia is recent as the patient has no collateral arteries on the CTA, High D-Dimers level, and a rapid response to unfractionated Heparin.

Treatment of neonatal spontaneous arterial thrombosis is controversial. An expert panel on the management of arterial thromboembolic events in neonates recommended that therapy should be individualized based on the extent of thrombosis and the urgency of the clinical situation [8], having anticoagulation agents as the recommended initial treatment for neonatal thromboembolism [7, 48–50] and thrombolytic agents reserved for selected cases where there is limb, organ, or neonatal life threatening event. Moreover, expectant management still has its success in selected cases [12, 51]. However, early diagnosis and prompt management are the essential parts for preserving limb function and perfusion as some cases were associated with a favorable outcome [3, 20, 22, 26]. There are no large trials comparing different therapeutic regimens [7, 52], making the decision highly individualized and based on clinical picture. 

We used Heparin followed by low-molecular-weight Heparin (Enoxaparin), the safest and most commonly used anticoagulant in neonatal thrombosis [7, 49, 53–55]. Heparin should be limited to clinically significant thrombosis with a goal of preventing clot expansion or embolism [7]. Heparin showed significant improvement without the need to thrombolytic therapy in our patient. This is probably as our patient had a “forming thrombus” rather than a “well formed thrombus” supported by having no collaterals found on CTA, low blood fibrinogen level, and a rapid improvement on anticoagulation alone. 

The safety of the low-molecular weight heparin (Enoxaparin) as an anticoagulant in newborns has been demonstrated [49], and it is indicated for the primary treatment of neonatal thromboembolism. Overall, LMWH has been effective in NICU setting with reports of partial or complete resolution of thromboembolic events in 59–100% of cases [53, 56–58]. Our patient was given Enoxaparin for 17 months. No consensus is present yet for the duration of treatment after resolution of symptoms. 

All neonates with thromboembolic disease should be evaluated for hypercoagulation, particularly those with spontaneous thrombosis in the absence of central catheters as described in our case. Consultation with a pediatric hematologist and a vascular surgeon is preferred in all cases. Neonatal ischemic stroke has also been reported in association with presence of one or more coagulation abnormalities. Gunther et al. have recommended a complete prothrombotic screening in all neonates with vascular accidents [43].

Recurrence can occur which is obvious from the inherited nature of the disease and the positive thrombophilia study of both parents. Increased rates of still birth, miscarriage, abruption of placenta, placental infarction, intrauterine fetal growth retardation, prematurity, and intrauterine fetal thrombosis were explained to the parents [11, 16, 59–61].

## 3. Conclusion 

This is one of the few reported cases of an unusual presentation of thrombophilia presenting at birth with intrauterine limb thrombosis successfully treated with Heparin and Enoxaparin. Both are noninvasive measures which proved to be effective. Regular followup showed no complication and no recurrence of a thrombus anywhere. To the best of our knowledge, this is the first reported case of an intrauterine brachial artery thrombosis which was successfully treated noninvasively with full recovery and no complications after a followup of 24 months.

## 4. Recommendations

We recommend investigating neonates and their parents for complete thrombophilia mutations when they present with unusual vascular occlusion site as newborns.

## References

[1] Kenny D, Tsai-Goodman B (2007). Neonatal arterial thrombus mimicking congenital heart disease. *Archives of Disease in Childhood*.

[2] Greenway A, Massicotte MP, Monagle P (2004). Neonatal thrombosis and its treatment. *Blood Reviews*.

[3] Aslam M, Guglietti D, Hansen AR (2008). Neonatal arterial thrombosis at birth: case report and literature review. *American Journal of Perinatology*.

[4] Sawyer T, Antle A, Studer M, Thompson M, Perry S, Mahnke CB (2009). Neonatal pulmonary artery thrombosis presenting as persistent pulmonary hypertension of the newborn. *Pediatric Cardiology*.

[5] Saxonhouse MA (2012). Management of neonatal thrombosis. *Clinics in Perinatology*.

[6] Manco-Johnson MJ, Grabowski EF, Hellgreen M (2002). Laboratory testing for thrombophilia in pediatric patients: on behalf of the Subcommittee for Perinatal and Pediatric Thrombosis of the Scientific and Standardization Committee of the International Society of Thrombosis and Haemostasis (ISTH). *Thrombosis and Haemostasis*.

[7] Sharathkumar AA, Lamear N, Pipe S (2009). Management of neonatal aortic arch thrombosis with low-molecular weight heparin: a case series. *Journal of Pediatric Hematology/Oncology*.

[8] Monagle P, Chalmers E, Chan A (2008). Antithrombotic therapy in neonates and children: American College of Chest Physicians evidence-based clinical practice guidelines (8th edition). *Chest*.

[9] Schmidt B, Andrew M (1995). Neonatal thrombosis: report of a prospective Canadian and international registry. *Pediatrics*.

[10] Nowak-Göttl U, Von Kries R, Göbel U (1997). Neonatal symptomatic thromboembolism in Germany: two year survey. *Archives of Disease in Childhood*.

[11] Boffa M, Lachassinne E (2007). Infant perinatal thrombosis and antiphospholipid antibodies: a review. *Lupus*.

[12] van Elteren HA, Veldt HS, te Pas AB (2011). Management and outcome in 32 neonates with thrombotic events. *International Journal of Pediatrics*.

[13] Andrew M, Monagle P, Brooker L (2000). *Arterial Thromboembolic Complications in Pediatric Patients, Thromboembolic Complications During Infancy and Childhood*.

[14] Khriesat W, Al-Rimawi H, Lataifeh I, Al-Sweedan S, Baqain E (2010). Intrauterine upper limb ischemia associated with fetal thrombophilia: a case report and review of the literature. *Acta Haematologica*.

[15] Armstrong AP, Page RE (1997). Intrauterine vascular deficiency of the upper limb. *Journal of Hand Surgery*.

[16] Andrew ME, Monagle P, deVeber G, Chan AK (2001). Thromboembolic disease and antithrombotic therapy in newborns. *Hematology*.

[17] Arshad A, McCarthy MJ (2009). Management of limb ischaemia in the neonate and infant. *European Journal of Vascular & Endovascular Surgery*.

[18] Carr MM, Al-Qattan M, Clarke HM (1996). Extremity gangrene in utero. *Journal of Hand Surgery*.

[19] Veldman A, Nold MF, Michel-Behnke I (2008). Thrombosis in the critically ill neonate: incidence, diagnosis, and management. *Vascular Health and Risk Management*.

[20] Nagai MK, Littleton AG, Gabos PG (2007). Intrauterine gangrene of the lower extremity in the newborn: a report of two cases. *Journal of Pediatric Orthopaedics*.

[21] Hensinger RN (1975). Gangrene of the newborn. A case report. *Journal of Bone and Joint Surgery A*.

[22] Özgenel GY, Akin S, Uysal A, Köksal N, Özcan M (2000). Gangrene of the upper extremity in the newborn. *European Journal of Plastic Surgery*.

[23] Dakouré PWH, Béogo R, Barro D, Somé DA, Cessouma R, Kambou T (2010). Intrauterine ischemia of the right upper limb and hemiface: a case report. *Chirurgie de la Main*.

[24] Aydin U, Ozgenel Y, Kanturk R (2010). Vacuum-assisted closure therapy in newborn gangrene. *Journal of Plastic, Reconstructive and Aesthetic Surgery*.

[25] Tridapalli E, Stella M, Capretti MG, Faldella G (2010). Neonatal arterial iliac thrombosis in type-I protein C deficiency: a case report. *Italian Journal of Pediatrics*.

[26] Nazer H, Abu Rajab A, Qaryouti S (1987). Neonatal limb gangrene and renal vein thrombosis. Case report with review of literature. *European Journal of Pediatrics*.

[27] Ricciardelli E, Morgan RF, Lin KY (1995). In utero brachial artery thrombosis: limb salvage with postnatal urokinase infusion. *Annals of Plastic Surgery*.

[28] Demirel G, Oguz SS, Celik IH (2011). Evaluation and treatment of neonatal thrombus formation in 17 patients. *Clinical and Applied Thrombosis/Hemostasis*.

[29] Turnpenny PD, Stahl S, Bowers D, Bingham P (1992). Peripheral ischaemia and gangrene presenting at birth. *European Journal of Pediatrics*.

[30] Uva MS, Serraf A, Lacour-Gayet F (1993). Aortic arch thrombosis in the neonate. *Annals of Thoracic Surgery*.

[31] Zeevi B, Berant M (1999). Spontaneous aortic arch thrombosis in a neonate. *Heart*.

[32] Nowak-Göttl U, Junker R, Kreuz W (2001). Risk of recurrent venous thrombosis in children with combined prothrombotic risk factors. *Blood*.

[33] Metsvaht T, Hermlin T, Kern H, Kahre T, Starkopf J (2006). Aortic arch thrombosis in a neonate with heterozygous carrier status of factor V leiden mutation. *Congenital Heart Disease*.

[34] Kosch A, Kuwertz-Bröking E, Heller C, Kurnik K, Schobess R, Nowak-Göttl U (2004). Renal venous thrombosis in neonates: prothrombotic risk factors and long-term follow-up. *Blood*.

[35] Kenet G, Sadetzki S, Murad H (2000). Factor V Leiden and antiphospholipid antibodies are significant risk factors for ischemic stroke in children. *Stroke*.

[36] Zenz W, Bodó Z, Plotho J (1998). Factor V Leiden and prothrombin gene G 20210 a variant in children with ischemic stroke. *Thrombosis and Haemostasis*.

[37] Nowak-Göttl U, Sträter R, Dübbers A, Oleszuk-Raschke K, Vielhaber H (1996). Ischaemic stroke in infancy and childhood: role of the Arg506 to Gln mutation in the factor V gene. *Blood Coagulation and Fibrinolysis*.

[38] Andrew M, David M, DeVeber G, Brooker LA (1997). Arterial thromboembolic complications in paediatric patients. *Thrombosis and Haemostasis*.

[39] Rees D, Cox M, Clegg JB (1995). World distribution of factor V Leiden. *The Lancet*.

[40] Dizon-Townson DS, Meline L, Nelson LM, Varner M, Ward K (1997). Fetal carriers of the factor V Leiden mutation are prone to miscarriage and placental infarction. *American Journal of Obstetrics and Gynecology*.

[41] Ridker PM, Miletich JP, Buring JE (1998). Factor V Leiden mutation as a risk factor for recurrent pregnancy loss. *Annals of Internal Medicine*.

[42] Ridker PM, Miletich JP, Hennekens CH, Buring JE (1997). Ethnic distribution of factor V Leiden in 4047 men and women: implications for venous thromboembolism screening. *Journal of the American Medical Association*.

[43] Gunther G, Junker R, Strater R (2000). Symptomatic ischemic stroke in in full term neonates: role of acquired and genetic prothrombotic risk factors. *Stroke*.

[44] Curry CJ, Bhullar S, Holmes J, Delozier CD, Roeder ER, Hutchison HT (2007). Risk factors for perinatal arterial stroke: a study of 60 mother-child pairs. *Pediatric Neurology*.

[45] Nowak-Göttl U, Koch HG, Aschka I (1996). Resistance to activated protein C (APCR) in children with venous or arterial thromboembolism. *British Journal of Haematology*.

[46] Hagstrom JN, Walter J, Bluebond-Langner R, Amatniek JC, Manno CS, High KA (1998). Prevalence of the factor V Leiden mutation in children and neonates with thromboembolic disease. *Journal of Pediatrics*.

[47] Nowak-Göttl U, Sträter R, Heinecke A (1999). Increased lipoprotein(a) is an important risk factor for venous thromboembolism in childhood. *Blood*.

[48] Ade-Ajayi N, Hall NJ, Liesner R (2008). Acute neonatal arterial occlusion: is thrombolysis safe and effective?. *Journal of Pediatric Surgery*.

[49] Edstrom CS, Christensen RD (2000). Evaluation and treatment of thrombosis in the neonatal intensive care unit. *Clinics in Perinatology*.

[50] Shama A, Patole SK, Whitehall JS (2002). Low molecular weight heparin for neonatal thrombosis. *Journal of Paediatrics and Child Health*.

[51] Bendaly EA, Batra AS, Ebenroth ES, Hurwitz RA (2008). Outcome of cardiac thrombi in infants. *Pediatric Cardiology*.

[52] Yurttutan S, Ozdemir R, Erdeve O (2012). Intrauterine upper extremity thrombosis successfully treated with recombinant tissue plasminogen activator, enoxaparin and collagenase. *Acta Haematologica*.

[53] Streif W, Goebel G, Chan AKC, Massicotte MP (2003). Use of low molecular mass heparin (enoxaparin) in newborn infants: a prospective cohort study of 62 patients. *Archives of Disease in Childhood*.

[54] Alioglu B, Ozyurek E, Tarcan A, Atac FB, Gurakan B, Ozbek N (2006). Heterozygous methylenetetrahydrofolate reductase 677C-T gene mutation with mild hyperhomocysteinemia associated with intrauterine iliofemoral artery thrombosis. *Blood Coagulation and Fibrinolysis*.

[55] Dix D, Andrew M, Marzinotto V (2000). The use of low molecular weight heparin in pediatric patients: a prospective cohort study. *Journal of Pediatrics*.

[56] Michaels LA, Gurian M, Hegyi T, Drachtman RA (2004). Low molecular weight heparin in the treatment of venous and arterial thromboses in the premature infant. *Pediatrics*.

[57] Malowany JI, Monagle P, Knoppert DC (2008). Enoxaparin for neonatal thrombosis: a call for a higher dose for neonates. *Thrombosis Research*.

[58] Malowany JI, Knoppert DC, Chan AKC, Pepelassis D, Lee DSC (2007). Enoxaparin use in the neonatal intensive care unit: experience over 8 years. *Pharmacotherapy*.

[59] Meinardi JR, Middeldorp S, De Kam PJ (1999). Increased risk for fetal loss in carriers of the factor V Leiden mutation. *Annals of Internal Medicine*.

[60] Kupferminc MJ, Eldor A, Steinman N (1999). Increased frequency of genetic thrombophilia in women with complications of pregnancy. *New England Journal of Medicine*.

[61] Metsvaht T, Hermlin T, Kern H, Kahre T, Starkopf J (2006). Aortic arch thrombosis in a neonate with heterozygous carrier status of factor V leiden mutation. *Congenital Heart Disease*.

